# Novel Strategies for Androgenetic Alopecia Therapy: Integrating Multifunctional Plant Extracts with Nanotechnology for Advanced Cutaneous Drug Delivery

**DOI:** 10.3390/pharmaceutics17091220

**Published:** 2025-09-19

**Authors:** Ruohan Diao, Meiqi Sun, Ningxin Zhang, Xinqian Liu, Ping Song

**Affiliations:** Department of Dermatology, Xiyuan Hospital of China Academy of Chinese Medical Sciences, Haidian District, Beijing 100091, China; ruohandiao@bucm.edu.cn (R.D.); sunmeiqi828@126.com (M.S.); znx00526@126.com (N.Z.); liuxinqian15980021819@bucm.edu.cn (X.L.)

**Keywords:** androgenetic alopecia, plant extracts, nanotechnology, skin delivery

## Abstract

Androgenetic alopecia (AGA), the most common form of hair loss, imposes considerable psychosocial and medical burdens. Current topical treatments are limited by suboptimal efficacy, slow onset, side effects, and poor patient adherence. Although numerous reviews have explored natural plant-based strategies for managing AGA, most offer fragmented evidence with limited systematic correlation between mechanistic studies and clinical outcomes concerning single plant constituents. This review critically synthesizes recent pharmaceutical advances in AGA therapy, with a focus on the synergistic potential of multifunctional plant extracts integrated with nanotechnology enhanced cutaneous delivery systems. We begin by examining the mechanistic basis of AGA pathogenesis and the limitations of existing treatments to identify unmet therapeutic needs. Next, we systematically evaluate plant extracts supported by robust in vitro, in vivo, and clinical evidence for their anti-androgenic, anti-inflammatory, antioxidative, and anti-apoptotic properties. Finally, we address key biopharmaceutical challenges in transdermal delivery for AGA and discuss how nanocarriers can overcome these barriers to improve local drug bioavailability and target specificity. By bridging phytochemistry and nanomedicine, this review provides novel insights and a pharmaceutics-oriented framework aimed at developing safer, more effective, and patient-compliant topical therapies for AGA.

## 1. Introduction

According to a survey conducted among residents aged 18 and above in six Chinese cities, the prevalence of AGA was 21.3% in men and 6.0% in women. In contrast, a study from Turkey reported considerably higher rates—67.1% in men and 23.9% in women. Among people of European descent, up to 80% of men and 40% of women show signs of AGA by the age of 70 or beyond. As a chronic condition, AGA not leads to cosmetic concerns but also substantially affects patients’ social perception, self-identity, and psychological well-being, contributing to a significant disease burden. Research indicates that individuals with AGA often experience psychological distress, including anxiety, feelings of helplessness, and reduced self-esteem. Importantly, such symptoms may be alleviated through active and appropriate treatment.

Hair is an important part of one’s external image. In today’s society, more and more people are troubled by hair loss. Androgenetic alopecia (AGA) is a common form of non-scarring alopecia, characterized by a higher incidence in men than in women and progressive worsening with age [1,2]. According to a survey conducted among residents aged 18 and above in six Chinese cities, the prevalence of AGA was 21.3% in men and 6.0% in women [3]. In a separate study from Turkey, the corresponding figures were markedly higher, at 67.1% and 23.9%, respectively [4]. Among individuals of European ancestry, by the age of 70 years or later, up to 80% of men and 40% of women exhibit clinical signs of AGA [2]. As a chronic disease, AGA not only causes disfiguring problems, but also has a significant adverse impact on patients’ social cognition, self-identity and mental health, leading to a heavy disease burden [5,6].

Current first-line management primarily involves topical pharmacotherapy. Topical minoxidil remains the only topical formulation approved by the U.S. Food and Drug Administration for AGA. Oral finasteride is widely used in male patients, whereas its topical formulation is a relatively recent approach and has not been globally standardized [2,7]. Although these therapies can slow disease progression or promote partial regrowth, Bayesian network meta-analyses indicated that there is limited strong evidence demonstrating their ability to significantly increase terminal hair counts. Furthermore, their efficacy typically peaks within 12 weeks and subsequently plateaus [8]. In addition to the limitations of therapeutic effects, the existing therapeutic drugs and administration methods also have disadvantages such as adverse drug reactions and low drug utilization rates. Exploring better treatment regimens for AGA is of great significance.

Recently, plant-derived extracts have gained increasing attention as potential therapeutic agents for AGA, owing to their multiple bioactive constituents capable of targeting diverse pathogenic pathways. Advanced nanocarriers can enhance transdermal delivery, improve active compound stability, and achieve targeted follicular deposition, potentially optimizing therapeutic efficacy while minimizing systemic exposure. However, existing reviews on phytotherapy for AGA are largely fragmented, often focusing solely on isolated in vitro, in vivo, or clinical studies. A distinct gap exists in systematic reviews that follow a translational pathway for the same plant constituent, from bench to bedside. Moreover, very few reviews have analyzed nanocarrier-based delivery systems from the standpoint of AGA-specific pathophysiology, particularly how they can overcome the drug delivery challenges inherent to this condition.

Therefore, this review aims to systematically integrate evidence on multifunctional plant constituents with robust mechanistic, preclinical, and clinical support, and to critically evaluate nanotechnology-enabled cutaneous delivery approaches for AGA. By positioning phytotherapy within a nanomedicine framework, we seek to provide a pharmaceutics-oriented perspective on developing safer, more effective, and patient-tailored interventions to address unmet clinical needs in AGA management.

## 2. Pathogenesis and Therapeutic Limitations

### 2.1. Follicular Architecture and Hair Follicle Cycling

**Figure 1:**
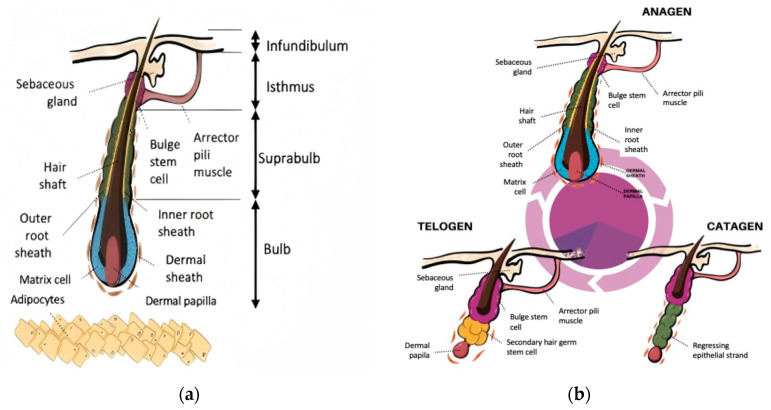
Hair follicle structure and cycling phases. (**a**) Anatomical structure of the hair follicle; (**b**) Key stages of the hair follicle cycle. (Reproduced from Cuevas-Diaz Duran R et al. [9], which is licensed under a Creative Commons Attribution-(CC BY 4.0) International License http://creativecommons.org/licenses/by/4.0/ accessed on 9 August 2025).

The hair follicle is a miniature organ composed of a variety of cells. In anatomy, the hair follicle is divided into four parts from top to bottom (Figure 1a) [9,10]. The epidermal opening of the hair follicle to the opening of the sebaceous gland is called the infundibulum, which is one of the important entrances for percutaneous drug absorption and can increase drug accumulation [11]. The region spanning from the opening of the sebaceous gland to the bulge is defined as the isthmus, with the bulge contains of a large number of hair follicle stem cells (HFSCs), tightly connected to the dermal papilla via the arrector pili muscle (APM). The transition from the attachment point of the APM to the top of the bulb is called the suprabulb. The hair bulb is located at the bottom of the hair follicle and is the “growth center” of hair, which is composed of melanocytes, hair matrix cells (HMCs) and dermal papilla (DP). DP is the “command center” of hair follicle, which can regulate the differentiation of HMCs and the hair follicle cycle [12,13]. HMCs surround the dermal papilla from the lateral and upper parts, where melanocytes are scattered and differentiated into hair shaft and inner root sheath under the stimulation of DP to form hair fibers [13,14,15]. In addition, the hair bulb to bulge of hair follicles is enclosed by dermal sheath, which provides an ecological niche for hair follicles and participates in hair follicle degeneration through smooth muscle contraction [16,17].

In the course of human life, hair follicles regenerate periodically (Figure 1b). The hair follicle cycle is divided into three stages: the anagen (2 to 8 years), the catagen (about 2 weeks), and the telogen (2 to 3 months) [18]. During the anagen, DP and secondary hair germs (SHG) located below the bulge interact, activate and proliferate under the drive of Wnt/β-catenin pathway. Meanwhile, hair follicles extend deep into the dermis [19,20,21], accompanied by an enlargement of the hair bulb, where HMCs exhibit rapid proliferation and differentiation, ultimately driving the continuous elongation of hair fibers [22]. In the late stage of anagen, overexpression of fibroblast growth factor 5 (FGF5) terminates the unlimited growth of hair, and hair follicles turn from the anagen to the catagen [23,24]. In the catagen, the β-catenin signaling is inhibited [25], while the transforming growth factor-β (TGF-β) signaling becomes predominant [26]. Proliferation of HMCs and DP cells arrest, followed by apoptosis, leading to a reduction in hair bulb volume [27]. In this stage, the dermal sheath continuously shrinks and compresses under the action of endothelin, pushing the hair shaft upward to prepare for hair loss. At the same time, the dermal sheath pulls the DP deep in the dermis upward to the underside of the bulge through the regressing epithelial strand [17,28], and connects with the SHG composed of HFSCs [29]. Subsequently, the hair follicles transitions into the telogen, during which the cell cycle of HFSCs is arrested, waiting for the reactivation of Wnt/β-catenin signaling pathway to initiate the next anagen [30,31]. During a person’s life, each hair follicle undergoes several cycles of hair follicles independently. There is sufficient research evidence that at any random time point, about 88–90% of the human scalp hair follicles are in the anagen, and 10–12% of the scalp hair follicles are in the catagen or telogen. Therefore, it is a normal physiological phenomenon that several hairs are lost from the hair follicles every day [32]. However, any changes in the hair follicle cycle, such as the depletion of HFSCs [33], abnormal hair follicle structure [34], and abnormal survival of the epithelial chain [35], can lead to pathological alopecia.

### 2.2. Pathogenesis of AGA

#### 2.2.1. Androgen Metabolism: Dihydrotestosterone-Driven Pathogenic Cascade in Hair Follicles

As the name suggests, androgens are a central factor in the pathogenesis of AGA. In the skin, androgens have physiological functions such as regulating hair growth, sebaceous secretion, and wound healing [36]. As the main circulating hormone in the blood, testosterone can be converted into 5α-dihydrotestosterone (DHT), a potent androgen with stronger affinity to androgen receptor (AR), by the action of 5α-reductase [37]. The combination of DHT and AR can inhibit the initial activation of Wnt signaling pathway, stimulate the expression of TGF-β signaling, and then induce apoptosis of microvascular endothelial cells and dermal papilla cells (DPCs), leading to miniaturization of hair follicles. The thick, hard terminal hair transforms into fine, soft vellus-like hair, and the anagen ends prematurely, eventually leading to alopecia [38,39]. Other studies have shown that AR expression is reduced in occipital hair follicles [40], while aromatase, which converts testosterone and DHT into estrogen, is increased in the forehead of women [41,42]. However, the consistency of these sex-specific expression patterns across different populations has not been fully established; variations in ethnicity, study design, and sample size may contribute to the heterogeneity reported in the literature, warranting further large-scale, multi-ethnic investigations. In addition to explaining the difference in hair loss pattern between men and women (male hair loss mainly concentrated in the frontotemporal and vertex, female hair loss showed diffuse thinning [43]), these results once again proved the importance of DHT and AR in the pathogenesis of AGA.

#### 2.2.2. Genetic Susceptibility: Polygenic Determinants of Follicular Vulnerability

AGA has a significant familial inheritance tendency, and several specific genomic loci contribute to the genetic susceptibility to the disease [44,45]. Based on available literature, Table 1 summarizes representative genes and loci reported to be associated with AGA in different populations. These include the AR and ectodysplasin A2 receptor genes on the X chromosome in European males [46,47]; the 20p11 locus, which appears more predictive in Asian males [48,49,50]; single nucleotide polymorphisms in ESR2 and CYP19A1 genes in females [51,52]; and polymorphisms in pathways such as Wnt, TGF-β, and hypoxia-inducible factor-1α (HIF-1α) that have been implicated across diverse populations [44]. While this summary is not exhaustive, it highlights key examples that illustrate the genetic heterogeneity of AGA.

#### 2.2.3. Microenvironment Deterioration: Multifactorial Perturbations in Inflammation, Microbiota and Microvasculature

In the process of AGA, the hair follicle microenvironment changes significantly. Histopathological evidence showed that inflammatory infiltration composed of lymphocytes and macrophages occurred in the infundibulum of hair follicles in the alopecia area of AGA patients, and this change also occurred in the non-alopecia area of AGA patients. The degree of inflammation in the non-alopecia area was milder than that in the alopecia area but significantly higher than that in the healthy control group. This suggests that hair follicle microinflammation in AGA may not merely be a secondary change; rather, it could act both as an active contributor—initiating and accelerating follicular miniaturization—and as a result of ongoing tissue remodeling. These observations support a bidirectional relationship between inflammation and AGA progression [53,54]. Genomic studies have found that natural killer cells, CD8+ T cells, mast cells and other inflammatory cells are significantly enrichment in AGA hair follicles [55,56], and the examination of blood biomarkers in AGA patients also supports the presence of inflammation [57]. Inflammatory mediators can inhibit the proliferation of HMCs and hair shaft growth, and induce the premature transition of hair follicles from the anagen phase to the catagen phase [58]. Microinflammation may also be involved in the pathogenesis of AGA by enhancing oxidative stress and inducing hair follicle cells apoptosis [59,60], but the specific mechanism needs to be further explored.

The scalp microbiota of AGA patients is different from that of healthy people, especially the increased abundance of *Propionibacterium acnes* and *Malassezia*, and the decreased abundance of *Corynebacterium*. This may be related to the stimulation of sebaceous gland secretion by DHT, which provides a lipid environment for the survival of bacteria [61,62,63]. Meanwhile, the microbiota and its metabolites may aggravate AGA by affecting hormone metabolism, triggering inflammatory response, and destroying skin barrier [62,64].

Blood supply is also a key factor to regulate the homeostasis of the microenvironment. In different hair follicle cycles, periodic vascular remodeling around the hair follicle is involved in regulating the activation and quiescence of HFSCs [65]. However, in AGA patients, DP in the hair loss area shows vascular degeneration in the early stage of hair follicle miniaturization [40], and this change can interfere with the normal hair cycle by reducing the supply of oxygen and nutrients to DP. Similarly, the expression of vascular endothelial growth factor (VEGF) is also decreased in alopecia area, which may enhance the apoptosis of HFSCs induced by DHT and participate in the process of hair follicle miniaturization [66]. Notably, several plant extracts have been reported to stimulate VEGF expression and promote perifollicular angiogenesis, thereby potentially counteracting these vascular changes. These strategies will be discussed in detail in later sections of this review.

#### 2.2.4. Cellular Dysfunction: Oxidative Damage and Senescence as Terminal Pathophysiology

Elevated markers of oxidative stress have been observed in the peripheral serum of patients with AGA [57], which may be related to the degeneration of hair follicle microvessels, microinflammation, and the inhibition of HIF-1α expression [67]. Compared with non-alopecia areas, DP in alopecia areas of AGA patients exhibits heightened sensitivity to oxidative stress [68], which has been confirmed to lead to apoptosis of HMCs, hair shaft growth retardation, and hair follicle proliferation in ex vivo culture experiments of human scalp hair follicles [69]. Oxidative stress-related DNA damage and mitochondrial dysfunction can accelerate the aging process of DPCs and HFSCs [70,71], and the increase in insulin-like growth factor-1 (IGF-1) with aging also contributes to this process [72]. Senescent hair follicle cells induce the senescence of neighboring cells by secreting senescence-associated secretory phenotypes, rapidly expanding the proportion of senescent cells, disrupting hair follicle homeostasis, depleting HFSCs, inhibiting hair-inducing properties, and eventually leading to hair follicle miniaturization and hair loss [72,73].

**Figure 2:**
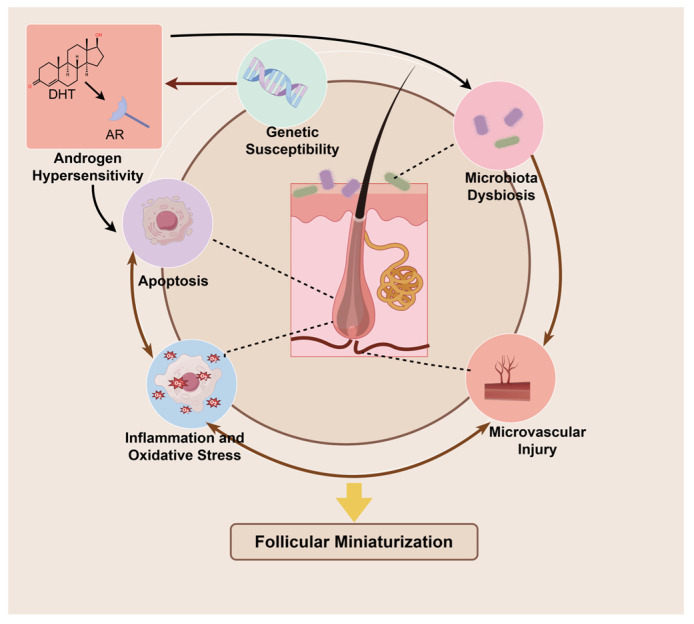
Pathogenic mechanisms of AGA.

In short, the pathogenesis of AGA is a complex interaction network with increased androgen sensitivity of hair follicles as the core, driven by genetic factors, which induces deterioration of hair follicle microenvironment and cell dysfunction, together leading to abnormal hair follicle cycle and hair follicle miniaturization (Figure 2). Other factors that cannot be listed one by one, such as smoking, diet, sleep, stress, prostaglandins, nutritional deficiency, lipid metabolism, are also involved [60,74]. Therefore, it is of great significance to find multi-target and multifunctional therapeutic drugs for the clinical management of AGA.

### 2.3. Topical Treatment of AGA

#### 2.3.1. Minoxidil

Topical application of 2% or 5% minoxidil is one of the few FDA-approved treatment options for AGA, and its mechanism of action is not fully understood. As a potassium channel opener, minoxidil can relax blood vessels and stimulate the secretion of VEGF to promote hair follicle angiogenesis [75,76]. Moreover, minoxidil can prolong the anagen phase of hair follicles by activating β-catenin signaling pathway, thereby achieving anti-hair loss effects [77]. However, the efficacy of minoxidil varies from person to person, and patients with low sulfotransferase enzyme activity in hair follicles show no efficacy of minoxidil treatment due to their inability to convert minoxidil to the active form [78]. Topical minoxidil is generally effective after 6 months of continuous use, but retrospective studies show that only 27.5% of patients achieve long-term use of minoxidil for more than 6 months. Poor efficacy, side effects such as scalp itching, increased dandruff, contact dermatitis, facial hirsutism, headache, etc., are the reasons for discontinuation of minoxidil [79]. This indicates that accurate screening of potential patients, scientific patient education, and reduction in drug side effects may improve patient compliance and enhance clinical efficacy in the future.

#### 2.3.2. Finasteride

Oral finasteride has been approved by the FDA for the treatment of male AGA, but adverse effects such as decreased libido, erectile dysfunction, and gynecomastia have limited its use, making topical application an alternative treatment option. Finasteride mitigates alopecia symptoms by irreversibly binding to 5α-reductase and preventing the conversion of testosterone to DHT. This medication is designed to reduce DHT production within hair follicles while minimizing alterations in serum DHT levels, and topical administration further reduces this interference compared with oral administration [80]. In terms of efficacy, oral finasteride and topical finasteride have similar efficacy, while topical finasteride has fewer side effects, mainly manifested as local skin itching, burning, and scalp erythema [81]. It should be pointed out that the research history of finasteride topical administration is relatively short, and there is a lack of long-term, large-scale real-world study data, so the understanding of its safety and efficacy may be insufficient.

Collectively, the current therapeutic paradigm for androgenetic alopecia (AGA) reveals a limited armamentarium of topical agents, characterized by suboptimal efficacy profiles—including delayed onset and modest response rates—coupled with suboptimal treatment experience that compromises adherence, and frequently observed localized adverse events. Thus, it is very necessary to develop new topical drug strategies.

## 3. Therapeutic Potential of Multifunctional Botanicals

Humans have a long history of treating diseases with plants, and the rich chemical components in plant extracts can provide a basis for the development of new drugs. Plant extracts with ethnomedicinal relevance have drawn global interest in AGA management. Regional research preferences—often favoring locally prominent or traditionally used plants—have contributed to considerable heterogeneity in the evidence base, while systematic investigations of individual plant extracts remain relatively scarce. Several natural plants with potential for AGA treatment have been reviewed in the literature [82,83,84,85], but not all of them are supported by comprehensive in vitro and in vivo research evidence, nor robust clinical data regarding their topical application.

To screen and evaluate the relevant literature in a systematic way, we used a multi-step search process. First, we carried out a broad search in the PubMed database with general keyword pairs such as “plant extract” and “alopecia”. After experts re-viewed the resulting articles and reviews, we selected a group of candidate plants that had a more complete and well-studied evidence background. Then, we performed a second round of focused searches for each of these plants using their specific names along with terms like “alopecia” or “hair” to fully gather both mechanistic and clinical studies. To avoid knowledge fragmentation and to select plant extracts with genuine potential for cutaneous delivery, this section, based on the aforementioned search results, will focus on therapeutic candidates supported by at least one clinical study (topical administration is preferred), of which the mechanisms have been explored through in vivo or in vitro experiments. The key active ingredients and multifunctional targets of these plant extracts will be introduced. Other plant extracts with less comprehensive or emerging evidence are briefly summarized, while many potentially relevant agents fall beyond the scope of this review.

Notably, this section centers on plant extracts rather than individual active constituents, a decision made with careful consideration. Given the current state of research, our understanding of specific bioactive compounds within many promising plants remains incomplete. Importantly, the therapeutic effects of these complex mixtures are likely attributable to the synergistic interactions among multiple components. Accordingly, while key active constituents and their potential mechanisms are discussed in the following sections, the primary emphasis remains on plant extracts and their inherent multi-component nature, with the aim of providing a more comprehensive reference for novel drug development.

### 3.1. Saw Palmetto Extract

Saw palmetto (SP), with the scientific name *Serenoa repens (W. Bartram) Small*, is a medicinal plant native to North America. As early as the 18th century, the Native Americans had used saw palm fruit to treat prostate diseases, which caused researchers to pay attention to the anti-androgen effect of SP and extend its application to the treatment of AGA. The main components of SP extract include fatty acids (e.g., oleic acid: ~221 mg/g, linoleic acid: ~36.7 mg/g, palmitic acid: ~58.6 mg/g) and phytosterols (e.g., β-sitosterol: ~1.67 mg/g, campesterol: ~0.53 mg/g) [86]. A liposterolic extract of SP (5 μg/mL) was confirmed to ameliorate the DHT-induced decrease in viability of HaCaT in vitro [87]. Furthermore, a study using a commercially available SP extract (2.5 mg/mL) showed that it could also VEGF secretion from human microvascular endothelial cells (HMVECs) and promotes in vitro tube formation, reduce the expression of DHT-induced 5-α reductase II, enhance the expression of β-catenin protein in DPCs and protects the vascular endothelium from oxidative stress [88]. These results suggest that SP can improve AGA hair follicle status by anti-androgen, activating β-catenin pathway, promoting angiogenesis, anti-inflammation, and anti-oxidative stress. In the DHT-induced AGA mouse model (*n* = 8), a five-week daily treatment with a 50% liposterolic extract of SP promoted hair follicle growth and eruption; reduced inflammatory cell content and TGF-β2 protein expression; and modulated apoptosis-related proteins by decreasing cleaved Caspase-3 and Bax while increasing Bcl-2 [87].

Similarly, topical application of SP extract has also achieved good clinical efficacy in clinical trials. This randomized, double-blind, placebo-controlled study showed that after 10 weeks of twice-daily topical application of an emulsion containing SP extract, the topical SP group had a significant increase in the number of hairs compared with the placebo group, with an increase of 27% and 14%, respectively, at the 50th week of the study [89]. A prospective, open, within-subject comparison study of 50 AGA patients showed that after 12 weeks of using concentrated serum or lotion containing SP extract, The average hair count and the average hair width of the patients were significantly increased. After 12 weeks of using SP lotion, the average hair count of the patients did not change significantly, but the number of vellus hair was significantly reduced, the number of terminal hairs was further increased, and the patient satisfaction was further improved. No subjects withdrew from the trial due to side effects [90]. Several randomized controlled trials also have confirmed the efficacy of oral SP extract in AGA patients. In a placebo-controlled trial, oral SP extract significantly improved hair density [91], whereas in an active-controlled trial, its efficacy was inferior to that of oral finasteride [92].

Overall, SP extract demonstrates multi-target actions—anti-androgenic, modulation of follicular microenvironment, and regulation of hair cycle—that are supported by in vitro, animal, and multiple clinical studies. While the current evidence is encouraging, it remains constrained by small sample sizes, short follow-up periods, and heterogeneity in formulations and delivery routes. Large-scale, long-term, and standardized clinical trials are warranted to confirm its efficacy and safety in AGA management and to identify the most appropriate dosage forms and administration strategies.

### 3.2. Cacumen Platycladi Extract

Cacumen Platycladi (CP) is the leaf of *Platycladus orientalis* (L.) *Franco* (formerly *Thuja orientalis* L.), which is widely distributed in China [93]. Traditional Chinese medical classics believe that CP has the effect of promoting hair growth and regenerating black hair. The main active components of CP are terpenes and flavonoids, whose levels vary significantly with the extraction technique. For essential oils, hydrodistillation yielded α-pinene (17.83%) as the major compound, while steam distillation produced α-cedrol (12.44%) as the predominant component [93]. For flavonoids, when using 75% ethanol for ultrasonic-assisted extraction, quercitrin (1.03–4.71 mg/g) is the most abundant compound, along with other constituents such as amentoflavone (0.56–1.07 mg/g) and hinokiflavone (0.62–1.03 mg/g) [94].

In the in vitro culture of DPCs, water extract of CP (0.25~1%) enhanced cell viability, decreased the expression of cell cycle arrest markers and cell senescence markers p21 and p16 at both mRNA and protein levels, and significantly increased the expression of anti-apoptotic protein survivin. For the Wnt/β-catenin signaling pathway, CP extract decreased the transcription of its inhibitor Dickkopf-1 (DKK-1) gene and increased the transcription of the downstream core transcriptional effector LEF-1 gene. In addition, CP extract also stimulated the expression of growth factors bFGF, FGF7, PDG and VEGF and enhanced tyrosine kinase signaling pathway [95]. Another experiment on DPCs confirmed that CP extract promoted cell cycle transition from G0/G1 to S phase by increasing cyclin D1, CDK2 and CDK4 proteins and decreasing P21 protein, and promoted β-Catenin accumulation, proliferation and migration of DPCs through Akt/GSK3β pathway phosphorylation [96]. Cell proliferation and scratch assay of HUVECs confirmed the ability of CP ethanolic extract (1.5 mg/g) to promote angiogenesis, suggesting that CP extract can stimulate hair growth by improving blood supply to hair follicles [97]. For the androgen induced AGA mouse model (*n* = 10), treatment with a 10% (*v*/*v*) solution of the n-hexane extract of CP can promote the growth and development of hair follicles and hair growth, increase the contents of SOD, CAT and GSH-PX in the skin lesions, and reduce the content of MDA to resist oxidative stress [98]. CP extract also reduced DHT levels in the skin the expression of 5α-reductase in the skin lesions without affecting the serum androgen levels [99]. For 6–7-week-old C57BL/6 mice with hair follicles synchronously in the telogen phase, treatment with both the hot water and steam-distilled extracts of CP accelerated the onset of anagen and prolonged its duration [100,101].

Regarding clinical efficacy, a randomized, double-blind, placebo-controlled study in 60 volunteers demonstrated that a 12-week external application of CP extract solution significantly increased the density of terminal hair [95]. In another randomized, double-blind, placebo-controlled trial involving 20 middle-aged male AGA patients, exosomes enriched with Ecklonia cava and CP extracts were injected every 15 days. After 16 weeks, hair density increased in the treatment group, but not in the placebo group. At the end of the study, patients in the treatment group had more satisfied self-perception with their hair than those in the placebo group [102]. In particular, some researchers have paid attention to the problem of UV damage in AGA hair: after UV irradiation, hair from alopecia areas of AGA patients was more prone to extensive scale curling than hair from non-alopecia areas, while hair from healthy people shows good tolerance to UV irradiation. Eight flavonoids isolated from CP extract were applied to the hair surface of AGA. After UV irradiation, the flavonoids derived from CP showed better hair protection effect than vitamin E cream and commercial conditioner, and reduced the content of melanin free radicals in hair [103].

Other studies focused on the active component monomer in CP. Topical application of cedrol, the main component of CP volatile oil, can induce hair follicles in the shaving area of C57BL/6 mice and Wistar rats to turn into the anagen, making hair longer and thicker, and some dosage groups showed better effects than the minoxidil-positive control group. The comparison results between different gender groups suggest that cedrol is more effective in promoting hair growth in female C57BL/6 mice, of which the specific mechanism needs to be further explored [104,105]. (7E)-7, 8-dehydroheliobuphthalmin, a lignan derivative identified as the main active ingredient in the n-hexane extract of CP, reduced DHT-induced DPCs death and AR and TGF-β protein levels in vitro and activated the Wnt/β-catenin signaling pathway [98].

Taken together, current in vitro, in vivo, and preliminary clinical studies suggest that CP extracts and their active constituents exert multiple beneficial actions on the follicular microenvironment in AGA. These multi-target activities have translated into encouraging clinical outcomes across different formulations. Nevertheless, the existing evidence base is limited by the small number and size of clinical trials, heterogeneity in intervention protocols and outcome measures, and incomplete elucidation of certain mechanistic pathways. Well-designed, large-scale studies with extended follow-up, alongside systematic evaluation of sex-specific responses and delivery routes, are warranted to firmly establish the therapeutic role of CP in AGA management.

### 3.3. Panax Ginseng/Red Ginseng Extract

Panax ginseng (PG) is the fleshy root of *Panax ginseng C. A. Mey*., whose unique ginsenosides have a wide range of pharmacological effects, and have sparked interest in the treatment of AGA with PG extract. PG extract (20 ppm) could stimulate the proliferation of the outer root sheath (ORS) keratinocytes and inhibit apoptosis induced by DKK-1, a Wnt/β-catenin pathway antagonist. This phenomenon may be related to the increased Bcl-2/Bax protein ratio. Similar results were observed in ex vivo culture of human HF organ, where PG extract reversed the increase in Bax mRNA levels triggered by DKK-1 treatment, promoted hair shaft elongation, and counteracted the growth inhibition of hair follicles induced by DKK-1 [106]. For human DPCs, PG extract at a concentration of 5 μg/mL can promote cell proliferation and stimulate VEGF expression [107]. Ginsenoside Re, the most abundant ginsenoside in PG extract, can restore the inhibited Wnt/β-catenin pathway by enhancing autophagy in DPCs, inhibit the transition from the anagen phase to the catagen phase of the human hair follicle organ model, and promote hair shaft growth [108], which has been confirmed in nude mice and suggests that ginsenoside Re may be related to the inhibition of TGF-β-induced ERK activation [109]. Ginsenoside Rb1 and Rd are also important components of PG extract. Topical application of ginsenoside Rb1 and Rd in C57BL/6 mice increased the expression of p63 protein in hair matrix and ORS, which is a key protein for promoting epidermal differentiation and hair follicle development, and enhanced the proliferative activity of hair follicles [110]. In addition to ginsenosides, gintonin, a lysophosphatidic acid receptor ligand from ginseng, can cause transient elevation of cytosolic Ca^2+^ levels in DPCs, induce cell proliferation and release VEGF, and stimulate hair growth in C57BL/6 mice at the hair follicle telogen, leading hair follicles to transition from early telogen to anagen phase [111]. In terms of clinical efficacy, a 24-week, randomized, triple-blind, controlled minoxidil study involving 32 AGA patients showed that topical application of herbal extracts solution containing PG extracts, biochanin A and acetyl tetrapeptide-3 improved the hair count and hair mass index of patients, of which the result was no significant difference compared with 3% minoxidil solution group, and no local adverse reactions were observed [112].

The steam-processed PG is known as red ginseng (RG). Compared with PG, some ginsenosides in RG changed during the stewing process, and some characteristic chemical products appeared [113,114], so the therapeutic effect in AGA may be slightly different. In the in vitro study, RG extract at concentrations of 100–400 μg/mL enhanced DPCs viability while inhibiting their apoptosis, as reflected by up-regulation of Bcl-2 expression and down-regulation of Bax expression. At the same time, RG extracts could induce GSK-3 inactivation by phosphorylation and increase the β-catenin protein content in DPCs [115]. The AR mRNA level in DPCs was up-regulated by DHT stimulation, and RG extract eliminated this effect, showing a certain anti-androgen potential [116]. As a maintenance factor of hair follicle telogen, the expression of BMP4 in DPCs was significantly inhibited by RG extracts, which may cause hair follicles to enter the anagen phase in advance. Ginsenoside Rb1, Rg1 and Re monomers present in both RG and PG extracts also showed the same effect [114,117]. RG extract increased the number of Ki-67-positive hair mother keratinocytes and eliminated the inhibition of proliferation induced by DHT in human hair follicle organ culture experiments in vitro [116]. RG extracts also stimulated ORS cell proliferation [117]. In addition, Rg3, as a unique component in RG extract, has been confirmed to stimulate VEGF signaling [118] and reduce DHT-induced ROS accumulation and cell senescence in DPCs [119]. For in vivo study, RG extracts induced premature transformation of hair follicles from catagen to anagen, stimulated hair growth, and promoted the expression of growth factors VEGF and IGF-1 in 7-week-old C57BL/6 mice. In addition, the expressions of β-catenin protein and Wnt target proteins cyclin D1 and cyclin E were increased, the activities of antioxidant enzymes GPx and SOD-2 were increased, and the phosphatidylinositol 3-kinase (PI3K)/Akt pathway was activated in the skin lesions after treatment with RG extracts [115,120]. In the testosterone-induced AGA mouse model, a 10% extract of RG also showed positive regulatory effects on hair regeneration and hair follicle growth cycle, and reduced abnormally elevated TGF-β signaling [121]. No clinical trial has evaluated topical RG extract in AGA. A small controlled study in women with AGA reported that oral RG extract, when combined with topical 3% minoxidil, resulted in greater improvement in hair density and thickness than minoxidil alone [122]. Although current evidence remains limited, these mechanistic findings suggest a potential—yet still speculative—role for RG extract in AGA management. Of note, clinical trials have demonstrated that topical application of RG extract significantly reduces skin sebum levels and effectively eradicates *Propionibacterium acnes* [123]. As noted earlier, this bacterium’s overgrowth may worsen perifollicular inflammation in AGA; thus, its eradication could plausibly aid in improving scalp health, although this remains speculative.

Current evidence indicates that PG and its active constituents exert multi-faceted effects relevant to AGA. Notably, studies employing human hair follicle organ culture offering a physiologically relevant ex vivo model that bridges basic mechanistic research and clinical translation more effectively than conventional cell lines. Animal studies corroborate these findings. Limited clinical results show improvements in hair count and quality comparable to minoxidil, with good tolerability. However, robust clinical trials evaluating RG in AGA are lacking, and its potential must therefore be inferred cautiously from relevant data and preliminary experimental results. Further research is needed to establish the therapeutic role and optimal use of PG in AGA.

### 3.4. Rice Bran Extract

Rice (*Oryza sativa* L.) is one of the three major food crops in the world, and rice bran (RB) is one of the by-products in rice processing. As a cheap and readily available ingredient, RB has a long history of being used for skin and hair care in Asia. The phytosterols (such as β-sitosterol, campesterol), tocotrienols, γ-oryzanol and other active ingredients in RB provide the material basis for the treatment of AGA, although their abundance and composition are known to differ substantially depending on the rice variety [124,125]. In DPCs, RB extract (0.125 mg/mL) significantly reduced the gene expression of 5-α reductases SRD5A1, SRD5A2 and SRD5A3, and reduced lipopolysaccharide (LPS) -induced NO release and hydrogen peroxid-induced MDA increase, reflecting the anti-androgen, anti-inflammatory and anti-oxidation effects. RB extract also activated Wnt/β-catenin and Sonic Hedgehog signaling pathways related to hair growth promotion, and promoted VEGF gene transcription in DPCs [126]. In terms of regulating hair follicle cycle, intervention with RB extract (30 μL/mL) increased the activity of alkaline phosphatase (ALP), an important indicator of DP hair follicle induction capacity, and induced fibrin expression in DPCs [127]. In LPS-induced inflammation model of RAW 264.7 mouse macrophages, RB extract (0.10–0.125 mg/mL) also showed anti-inflammatory effect by inhibiting the release of NO [126,128]. Notably, RB extract has been shown to stimulate melanin production in B16F10 melanoma cells, thereby demonstrating its potential to enhance hair pigmentation. This effect may be related to its content of iron, zinc, and unsaturated fatty acids, which can modulate hair follicle pigmentation and whose deficiencies are linked to reversible graying [129]. In vivo, topical application of a 3% rice bran supercritical CO_2_ extract induced the premature activation of telogen-phase hair follicles in C57BL/6 mice (*n* = 5), the formation of DP and ORS structures, the deep penetration of hair roots into the subdermis, and accelerated hair growth. Increased VEGF and IGF-1 gene levels and decreased TGF-β gene levels again confirmed the hair growth promoting ability of RB. Further animal experiments confirmed the effectiveness of the components of RB extract including linoleic acid, pritol, γ-oryzanol, and γ-tocotrienol [130].

The efficacy of RB extract has been confirmed in clinical studies as well. In a 16-week, randomized, double-blind, placebo-controlled study involving 50 AGA patients, topical treatment with RB extract significantly increased mean hair density to 106% of baseline in male patients in the treatment group, whereas no significant change was observed in the placebo group. In female patients, there was a significant increase in average hair density in the treatment group, but it was not statistically different from that in the placebo group. This suggested that external coating of RB extract has some clinical efficacy, but it may not be significant in female AGA patients, and further clinical trials are needed to confirm this finding [131].

From the available evidence, RB extract and its active components demonstrate multi-target activities relevant to AGA. Randomized controlled trials indicate that topical RB extract can increase hair density in AGA, with some variation between sexes, though further large-scale, long-term studies are needed to confirm efficacy and optimize formulation and delivery strategies.

Table 2 summarizes the main active ingredients, mechanisms of action, and relevant details for the plants mentioned above.

In addition to the above plants, silybum marianum [134,135], rosemary [136,137,138,139], forsythiasis [140,141] and other plants also have certain therapeutic potential for AGA, but the evidence remains limited or a comprehensive in vitro–in vivo–clinical evidence chain has yet to be established (as summarized in Table 3). Therefore, this article does not review studies about these plants, and we look forward to more research evidence for reference in the future.

## 4. Nanodelivery Systems: Overcoming Therapeutic Barriers in AGA

Previous studies have proved that plant extracts have full potential for AGA treatment, but the process of delivering drug components to their targets is still a struggle against the complex physiological environment of hair follicles. First, the molecular characteristics of drugs, such as poor solubility and unstable chemical properties, greatly weaken the ability of drugs to take effect. Secondly, the skin barrier, including the mildly acidic pH, the lipid-rich sebum film, and the highly ordered stratum corneum, acts as a defensive fortification that markedly reduces the transdermal and transfollicular delivery efficiency of drugs. Finally, due to frequent administration and long course of treatment, traditional therapy easily causes patients to lose confidence in treatment and have poor compliance. In the face of these difficulties, nanocarriers offer opportunities to optimize the topical delivery system, including improvements in drug loading, targeted delivery, controlled release, and other aspects. This enables more accurate, efficient, and convenient treatment strategies. This section will introduce the challenges and solutions of skin-based drug delivery for AGA treatment.

### 4.1. Molecular Challenges: Nanoencapsulation Strategies

The natural active ingredients in plant extracts exhibit a broad spectrum of pharmacological effects. However, with low water solubility and easy degradation, plant extracts have limited absorption efficiency, preventing them from achieving desirable therapeutic efficacy. For instance, apigenin exhibits extremely low aqueous solubility (0.62 ± 0.88 μg/mL), yet when encapsulated in phytosome, its solubility increases approximately 35-fold, along with a significant improvement in antioxidant efficacy observed in rats [142]. Similarly, curcumin, which is prone to rapid degradation under physiological conditions, can be stabilized via incorporation into polymeric nanoparticles, resulting in enhanced chemical stability and therapeutic performance [143]. Nanotechnology can increase the solubility and stability of drugs by reducing particle size, providing lip-based system or polymer system, reducing the crystal precipitation of drugs on the surface of the skin, and reducing the skin irritation caused by traditional organic solvents such as ethanol and propylene glycol [144].

Nanoemulsion is a kind of dispersive system composed of hydrophobic dispersive phase, hydrophilic continuous phase and surfactants or stabilizers. The poorly soluble drug is encapsulated in nano-sized particles by the dispersed phase, which is evenly dispersed in the continuous phase, and the absorption and efficacy of the drug are enhanced by increasing the surface area-to-volume ratio [145]. Some self-developed rosemary [146], luteolin [147] and cedrol [148] drug-loaded nanoemulsions have been confirmed to improve the apparent solubility of drugs, and their stability was further verified by long-term storage and repeated freeze–thaw experiments. Similarly, the favorable efficacy of the nanoemulsion against AGA was also confirmed in model mice.

Liposome, solid lipid nanoparticles and nanostructured lipid carriers are commonly used lipid-based nanoparticles. They encapsulate drug molecules within phospholipid bilayers, solid lipids, or liquid oils, thereby enhancing drug-loading capacity and stability [149]. Based on the above carriers, nano-delivery systems have been developed for plant extracts such as tea seed oil [150], saw palmetto [151], and cardamonin [152], all of which show good stability and solubility, and show certain therapeutic potential for AGA in vivo or in vitro experiments.

Micelle is a nanostructure formed by spontaneous assembly of amphiphilic molecules or polymers in aqueous solution. The hydrophobic core of micelle is formed by the hydrophobic tail of amphiphilic molecules, which builds a tiny region isolated from the surrounding water environment and emplaces insoluble drug molecules here to protect them from the destruction of enzymatic hydrolysis, hydrolysis, oxidation and other environments. The hydrophilic shell is formed by the outward arrangement of the hydrophilic heads of the amphiphilic molecules, allowing the entire micelle to be stably dispersed in the aqueous solution [153,154]. A glycyrrhizin-baicalin co-loaded nanocarrier ingeniously leverages this characteristic of micelle: Baicalin has poor water solubility and low skin permeability, but glycyrrhizic acid is amphiphilic, so baicalin can be encapsulated in glycyrrhizic acid to form nano-micelles. In addition to improving drug loading and permeability, the combined nano-micelles also show better AGA treatment effect than either of them alone in vivo and in vitro experiments [155].

Hydrophilic and hydrophobic phytoconstituents in plant extracts are often difficult to compatibly formulate in a single dosage form, which poses a major obstacle to the simultaneous transdermal delivery of multi-target agents required for AGA therapy. Nanocarriers solve this problem by co-encapsulation technology: liposomes can encapsulate hydrophilic molecules in their core and embed hydrophobic molecules in the lipid bilayer. Polymeric Nanocarriers can form core–shell structures in aqueous dispersions. Dendrimers, with their hierarchical architecture, enable co-encapsulation by encapsulating or adsorbing drug components with distinct properties [156]. While co-encapsulation has been extensively explored in oncology, its application to AGA remains limited. A notable example is the previously described glycyrrhizin–baicalin micelle system, which was specifically designed for AGA treatment.

### 4.2. Cutaneous Barrier Penetration: Precision Follicular Targeting

The paracellular route, transcellular route and transfollicular route are three possible pathways in the process of transdermal drug absorption [157]. A study examining the topical application of caffeine to the human chest revealed that the maximum systemic bioavailability of the drug was predicted to be 20% higher when hair follicles were open compared to when they were obstructed [158]. Compared to the chest skin, the scalp exhibits a higher density of hair follicle distribution, and considering that hair follicles are the main lesion site of AGA, the hair follicle pathway is an important way of drug absorption in the local treatment of AGA.

The hair follicles open on the skin surface and are keratinized only at their initial parts. The keratinocytes at the lower end of the infundibulum are more permeable, so the keratinocyte barrier of the hair follicles is not as tight as the interfollicular skin [159,160]. Nanocarriers, by virtue of their size advantage, readily exploit the natural entry point of hair follicle openings to penetrate deep into the follicular structure, with their deposition sites varying within the follicle depending on particle size [161]. In a topical application study of retinol nanoparticles, the rapid penetration of retinol nanoparticles in hair follicles was visualized by fluorescent labeling, and retinol nanoparticles were demonstrated to have higher concentration and deeper skin accumulation than free retinol by differential stripping experiments [162]. In the study of treating AGA model mice with cedrol nanoemulsion, compared with free cedrol, the cedrol nanoemulsion also showed deeper hair follicle accumulation, and the nanoemulsion could further promote the rapid penetration of encapsulated drugs by changing the orderly structure between keratinocytes and lipids, which greatly shortened the onset time of drug in the treatment of AGA model mice [148].

**Figure 3:**
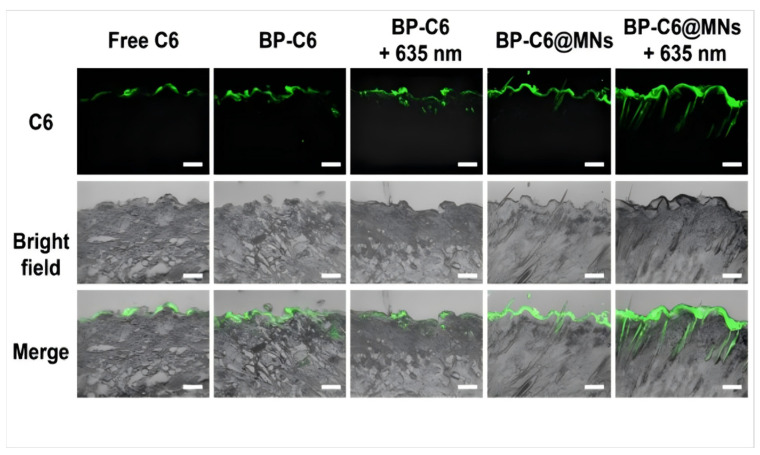
Enhanced transdermal drug delivery and follicular targeting through nanocarrier systems combined with penetration-promoting technologies. Confocal laser scanning microscope images of C6 permeation through the rat skin after 4 h treatment with different formulations. Scale bar: 200 μm. C6: coumarin 6 mimicked drug for visualization; BP-C6: black phosphorus nanosheets encapsulating C6; BP-C6 + 635 nm: BP-C6 with 635 nm laser irradiation; BP-C6@MNs: C6-loaded black phosphorus nanosheets encapsulated microneedles; BP-C6@MNs + 635 nm: BP-C6@MNs with 635 nm laser irradiation. (Reproduced from Xiong S et al. [165]. which is licensed under a Creative Commons Attribution-(CC BY 4.0) International License http://creativecommons.org/licenses/by/4.0/ accessed on 9 August 2025).

Compared with healthy people, AGA patients have the characteristics of prolonged hair follicle telogen phase and miniaturization of hair follicles, resulting in decreased hair follicle activity, decreased blood circulation, and weakened drug absorption efficiency. In the late AGA stage, yellow dots can be observed under trichoscopy, suggesting that some of the hair follicle openings have been blocked by keratin and/or sebum, and the drug absorption pathway is further blocked [163]. Therefore, nanodelivery systems are often employed in combination with various penetration-enhancing techniques to achieve higher drug delivery efficiency. For example, nanostructured lipid carriers loaded with β-sitosterol are integrated into chitosan-based microneedle, and the microneedle is used to penetrate the skin to deliver drugs into the deep dermis. Through microneedle treatment, the transdermal permeability of nano-lipid carrier can be effectively improved and the number of hair follicles in the anagen phase can be significantly increased [164]. Furthermore, BP-BA@MNs, a microneedle patch containing black phosphorus nanosheets loaded with baicalin, can induce photothermal effects secondary to 635 nm laser irradiation, showing higher dermal permeability and hair follicle accumulation than those treated with drug-loaded microneedle or drug-loaded nanosheets combined with laser irradiation (Figure 3), which offset the abnormal expression of key genes and altered hair growth cycle of DHT-induced AGA mice models [165].

### 4.3. Adherence Enhancement: Controlled-Release Platforms

A number of studies have shown that AGA patients often exhibit poor adherence to topical minoxidil treatment, frequently discontinuing the therapy prematurely before achieving noticeable therapeutic outcomes [79,166,167]. This result may be related to the high frequency of administration of minoxidil solution (standard usage is twice a day [168]) and the relatively slow effect, which causes inconvenience to patients’ life and fails to establish patients’ confidence in treatment. New drugs developed based on nano-delivery systems can make hair follicles obtain effective drug concentration faster by improving drug bioavailability, shorten the onset time, and reduce the early withdrawal caused by too long waiting period. At the same time, nanoparticles accumulate deep in the hair follicle opening to form a drug reservoir, which prolongs the retention time of the drug in the body and releases the drug for long-term and efficient drug delivery, making it possible to reduce the frequency of drug administration [169,170]. In the BP-BA@MNs trial described above, there was no significant difference in treatment effect between the intervention with BP-BA@MNs every 3 days and the intervention with minoxidil once daily.

In addition, some responsive intelligent carriers represented by surface-capped mesoporous silica nanoparticles can trigger the controlled release of drugs through physical or chemical environmental changes such as temperature and pH [171,172]. This provides the possibility of precise and individualized treatment for AGA patients.

## 5. Conclusions and Future Trajectories

Considering the high prevalence, negative psychosocial impact, and lack of efficacy and compliance of existing treatment options of AGA, it is imperative to develop new clinical treatment options for AGA. Plant extracts such as SP and CP provide some potential alternatives to traditional AGA treatment by simultaneously targeting multiple pathological processes such as androgen signaling, inflammation, oxidative stress, and apoptosis. In addition, advanced nanocarriers such as microemulsions, liposomes, nano-lipid carriers, micelles effectively address key limitations in the topical treatment of AGA, including poor solubility, insufficient hair follicle penetration, and frequent dosing, resulting in improved therapeutic efficacy and patient compliance. The combination of phytopharmacology and nanotechnology not only provides a mechanistic basis for multi-targeted interventions, but also lays the foundation for the design of entirely new AGA management options. To fully explore the therapeutic potential of related plants, future studies should consider: (1) Systematically screen and evaluate the multiple active components in plant extracts, further investigate their molecular effects, and decipher the multi-component synergistic mechanism of plant extracts; (2) Design high quality clinical research, carry out large sample, multi-center, long-term follow-up clinical controlled trials, and strictly evaluate the clinical efficacy and safety of plant extracts; (3) Optimize the formulation and fabrication of nanocarriers to enable the development of more efficient systems.

However, several translational and regulatory considerations must be addressed before these technologies can be widely adopted in clinical practice. Formulation stability under real-world storage and transport conditions, batch-to-batch consistency, and scalability of manufacturing processes remain key technical hurdles, as these factors directly impact the ability to control drug release kinetics and formulation optimization. Regulatory scrutiny is heightened due to unique nanomaterial characterization requirements and unresolved long-term biodistribution and toxicological profiles. Furthermore, potential immunogenicity and high production costs may hinder patient adherence, despite the potential for improved efficacy. Addressing these practical challenges through coordinated preclinical, clinical, and industrial efforts will be essential to translate the promise of nanotechnology-enabled therapies for AGA into safe, effective, and accessible treatment options.

Although challenges remain, the integration of plant extracts with multi-target therapeutic potential and advanced nano-delivery technology offers a promising and innovative path to address the existing therapeutic pain points of AGA. Through continued basic research breakthroughs, rigorous clinical validation, and interdisciplinary collaboration, this strategy is expected to lead to the development of safer, more efficient, more convenient, and better patient adherence to the management of AGA, and ultimately improve the quality of life of millions of patients worldwide.

## Figures and Tables

**Table 1:** Representative genetic loci associated with AGA in different populations.

Population	Gene/Locus	Function or Pathway	Reported Association Features	Reference(s)
European males	AR (X chromosome)	Regulates DHT sensitivity	Sequence variants may enhance follicular sensitivity to DHT, leading to earlier onset	[46,47]
	Ectodysplasin A2 receptor (X chromo-some)	Involved in ectodermal development and hair follicle morphogenesis	Variants associated with increased susceptibility to AGA	[46]
Asian & African males	X-linked loci	–	Association with AGA not significant in most studies	[48,49,50]
Asian males	20p11 locus	Autosomal	Shows higher predictive value for AGA; mechanisms remain unclear	[49,50]
Females	ESR2	Estrogen receptor	SNPs associated with AGA risk	[52]
	CYP19A1	Aromatase (converts androgens to estrogens)	SNPs associated with susceptibility	[51,52]
Multiple populations	Wnt pathway genes	Hair follicle growth regulation	Polymorphisms linked to AGA susceptibility	[44]
	TGF-β pathway genes	Hair follicle regression signaling	Variants associated with AGA	[44]
	HIF-1α	Hypoxia response pathway	Associated with AGA risk in some studies	[44]

**Table 2:** Natural plant extracts with potential for AGA treatment.

		Saw Palmetto	Cacumen Platycladi	Panax Ginseng	Red Ginseng	Rice Bran
Main Bioactive Components		Oleic acid, linoleic acid, palmitic acid, campesterol, β-sitosterol, etc.	α-pinene, α-cedrol, quercitrin, amentoflavone, hinokiflavone (7E)-7,8-Dehydroheliobuphthalmin, etc.	Gintonin, ginsenoside Re, Rb1, Rg1, Rd, etc.	Ginsenoside Re, Rb1, Rg1, Rg3, etc.	β-sitosterol, campesterol, tocotrienols, γ-oryzanol, etc.
In Vitro Studies		HaCaT, HMVECs, DPCs	DPCs, HUVECs	ORS keratinocytes, human HF organ, DPCs	DPCs, human HF organ, OSR cells	DPCs, RAW 264.7, B16F10
In Vivo Studies		DHT-Induced AGA mouse model	Androgen induced AGA mouse model, C57BL/6 Mice, Wistar rats	Nude mice, C57BL/6 mice	C57BL/6 mice, testosterone-induced AGA mouse model	C57BL/6 mice
Clinical Research Evidence (Topical Application) ^1^		Randomized, double-blind, placebo-controlled study; Prospective, open-label, self-controlled study	Randomized, double-blind, placebo-controlled study	Randomized, triple-blind, controlled minoxidil study		Randomized, double-blind, placebo-controlled study
Mechanism of Action	Anti-Androgenic	5-α reductase II↓ ^2^	AR↓, DHT↓, 5α reductase↓		AR↓	SRD5A1↓, SRD5A2↓, SRD5A3↓
	Anti-Inflammatory	Inflammatory cells↓				NO↓
	Antioxidative	Lipid peroxidation↓	SOD↑, CAT↑, GSH-PX↑, MDA↓		GPx↑, SOD-2↑, ROS↓	MDA↓
	Anti-Apoptotic	Cleaved caspase 3↓, Bcl-2↑, Bax↓	Survivin↑	TUNEL-positive cells↓, Bcl-2↑, Bax↓	Bcl-2↑, Bax↓	
	Pro-Proliferative	Cell viability↑	Cell viability↑	Cell viability↑	Cell viability↑, Ki67↑	
	Pro-Angiogenic	VEGF↑	Scratch assay (+)	VEGF↑	VEGF↑	VEGF↑
	Anti-Aging		p21↓, p16↓		β-Gal-positive cells↓	
	Modulation of the Microbiota				Cutibacterium↓	
	Signaling Pathways	Wnt/β-catenin pathway↑, TGF-β pathway↓	Wnt/β-catenin pathway↑, tyrosine kinase signaling pathway↑, Akt/GSK3β pathway↑, TGF-β pathway↓	Wnt/β-catenin pathway↑, TGF-β pathway↓	Wnt/β-catenin pathway↑, PI3K/Akt pathway↑, TGF-β pathway↓	Wnt/β-catenin pathway↑, Sonic Hedgehog pathway↑, TGF-β pathway↓
	Others		Promotes cell cycle progression from G0/G1 phase to S phase	p63↑, transient elevation of cytosolic Ca^2+^ levels	IGF-1↑, BMP4↓	ALP↑, fibronectin↑, IGF-1↑, melanin synthesis↑
Ref.		[87,88,89,90,91,92,132,133]	[93,94,95,96,97,98,99,100,101,102,103,104,105]	[106,107,108,109,110,111,112]	[113,114,115,116,117,118,119,120,121,122,123]	[124,125,126,127,128,129,130,131]

^1^ Detailed clinical trial designs, endpoints, and outcomes for each botanical listed are described in the corresponding sections of the main text; ^2^ ↑, upregulation; ↓, downregulation.

**Table 3:** Representative natural plant extracts with potential for AGA treatment (preliminary/incomplete evidence) ^1^.

	Silybum Marianum	Rosemary	Forsythiasis
Main Bioactive Components	Silibinin, apigenin, etc.	Rosmarinic acid, etc.	Forsythiaside-A, etc.
Study types	In Vitro (DPCs)/randomized, double-blind, placebo-controlled clinical trial (shampoo, rinse-off).	In Vivo (Wistar rats, C57BL/6 mice)/randomized, active-controlled clinical trial (topical)	In Vitro (DPCs, HaCaT)/In Vivo (C57BL/6 mouse)
Mechanism of Action	Pro-Proliferative, Pro-Angiogenic, Antioxidative, Anti-Aging, Wnt/β-Catenin pathway↑ ^2^, Akt pathway↑	Anti-Androgenic, Wnt/β-Catenin pathway↑	Anti-Apoptotic, TRPV3 pathway↓
Major clinical outcomes	Hair count↑	Hair count not significantly different from 2% minoxidil at 6 months	No clinical data
Ref.	[134,135]	[136,137,138,139]	[140,141]

^1^ These plants are not reviewed in detail in the main text. The evidence base is preliminary or incomplete (e.g., mainly in vitro/animal data or limited clinical findings), and the table lists representative examples only, not an exhaustive set; ^2^ ↑, upregulation; ↓, downregulation.

## Data Availability

Not Applicable.

## Abbreviations

The following abbreviations are used in this manuscript:

AGA	Androgenetic alopecia
ALP	Alkaline phosphatase
AR	Androgen receptor
CP	Cacumen Platycladi
DHT	5α-dihydrotestosterone
DKK-1	Dickkopf-1
DP	Dermal papilla
DPCs	Dermal papilla cells
FGF5	Fibroblast growth factor 5
HFSCs	Hair follicle stem cells
HIF-1α	Hypoxia inducible factor-1α
HMCs	Hair matrix cells
HMVECs	Human microvascular endothelial cells
HUVECs	Human umbilical vein endothelial cells
IGF-1	Insulin-like growth factor-1
LPS	Lipopolysaccharide
ORS	Outer root sheath
PG	Panax ginseng
PI3K	Phosphatidylinositol 3-kinase
RB	Rice bran
RG	Red ginseng
SHG	Secondary hair germ
SP	Saw palmetto
TGF-β	Transforming growth factor-β
VEGF	Vascular endothelial growth factor
